# Pityriasis Lichenoides et Varioliformis Acuta Associated With Tirzepatide: A First Report

**DOI:** 10.1111/1753-0407.70233

**Published:** 2026-06-17

**Authors:** Muzaffer Serdar Deniz, Gulhan Aksoy Sarac, Burak Celik, Merve Kaya, Hilal Balta, Muzeyyen Kecik, Oya Topaloglu, Reyhan Ersoy, Bekir Cakir

**Affiliations:** ^1^ Department of Endocrinology and Metabolism Diseases Ankara Bilkent City Hospital Ankara Turkiye; ^2^ Department of Dermatology Ankara Bilkent City Hospital Ankara Turkiye; ^3^ Department of Internal Medicine John H. Stroger, Jr. Hospital of Cook County Chicago Illinois USA; ^4^ Department of Pathology University of Health Science, Ankara Bilkent City Hospital Ankara Turkey; ^5^ Department of Endocrinology and Metabolism Diseases Ankara Yildirim Beyazit University, Faculty of Medicine Ankara Turkiye

Dear Editor,

1

Tirzepatide, a dual glucose‐dependent insulinotropic polypeptide (GIP) and glucagon‐like peptide‐1 (GLP‐1) receptor agonist, has demonstrated substantial efficacy in glycemic control and weight reduction in type 2 diabetes mellitus and obesity. Its overall safety profile is favorable, with gastrointestinal symptoms and injection‐site reactions representing the most commonly reported adverse events [1]. However, rare immune‐mediated dermatologic reactions continue to emerge with broader clinical use. We report, to our knowledge, the first case of pityriasis lichenoides et varioliformis acuta (PLEVA) temporally associated with tirzepatide therapy.

A 41‐year‐old man with type 2 diabetes mellitus (HbA1c 6.8%, BMI 30.5 kg/m^2^) developed a localized cutaneous eruption 5 days after his second weekly tirzepatide dose. He denied recent infections, systemic symptoms, or exposure to new medications aside from tirzepatide. Physical examination revealed clustered erythematous, mildly scaly papules on the right lateral tibial region, the largest measuring approximately 6–8 mm in diameter with a central hemorrhagic crust. There was no mucosal involvement, lymphadenopathy, or systemic findings. Laboratory evaluations, including complete blood count, complete metabolic panel, and lipid profile, were unremarkable except for an elevated fasting glucose of 127 mg/dL. Of note, lesions were confined to the right leg; while unusual, localized presentations of PLEVA have been documented.

Punch biopsy demonstrated mild spongiosis and acanthosis, minimal vacuolar degeneration of the basal layer, intraepidermal lymphocytic exocytosis, scattered neutrophilic debris, erythrocyte extravasation, and perivascular and lichenoid lymphocytic infiltration with melanophages in the dermis (Figure 1). Perivascular and periadnexal lymphocytic infiltration was also observed in the deep dermis (Figure 2). These findings argued against guttate psoriasis and lichen planus and were consistent with PLEVA [2]. Pityriasis rosea was excluded by the absence of a herald patch, and syphilis serology was negative. Lymphomatoid papulosis remained the closest histopathological mimicker; CD30 immunostaining and the clinical course are typically used to distinguish between them, though immunohistochemistry was not performed in this case. Following dermatological consultation, the patient opted to discontinue tirzepatide.

**FIGURE 1:**
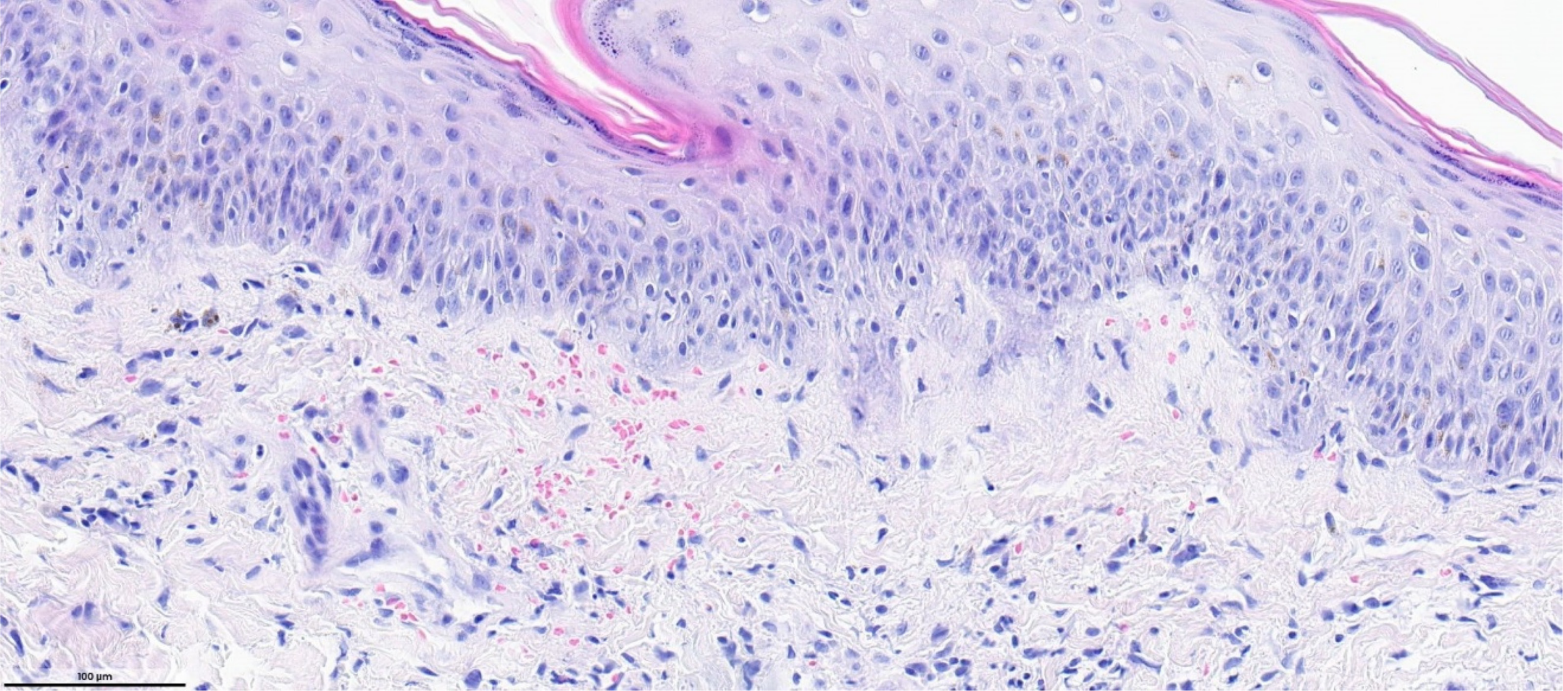
Mild spongiosis and acanthosis in the epidermis, along with minimal vacuolar degeneration in the basal layer and intraepidermal lymphocytic exocytosis. Scattered neutrophilic debris, erythrocyte extravasation, perivascular and lichenoid lymphocytic infiltration, and melanophages in the dermis.

**FIGURE 2:**
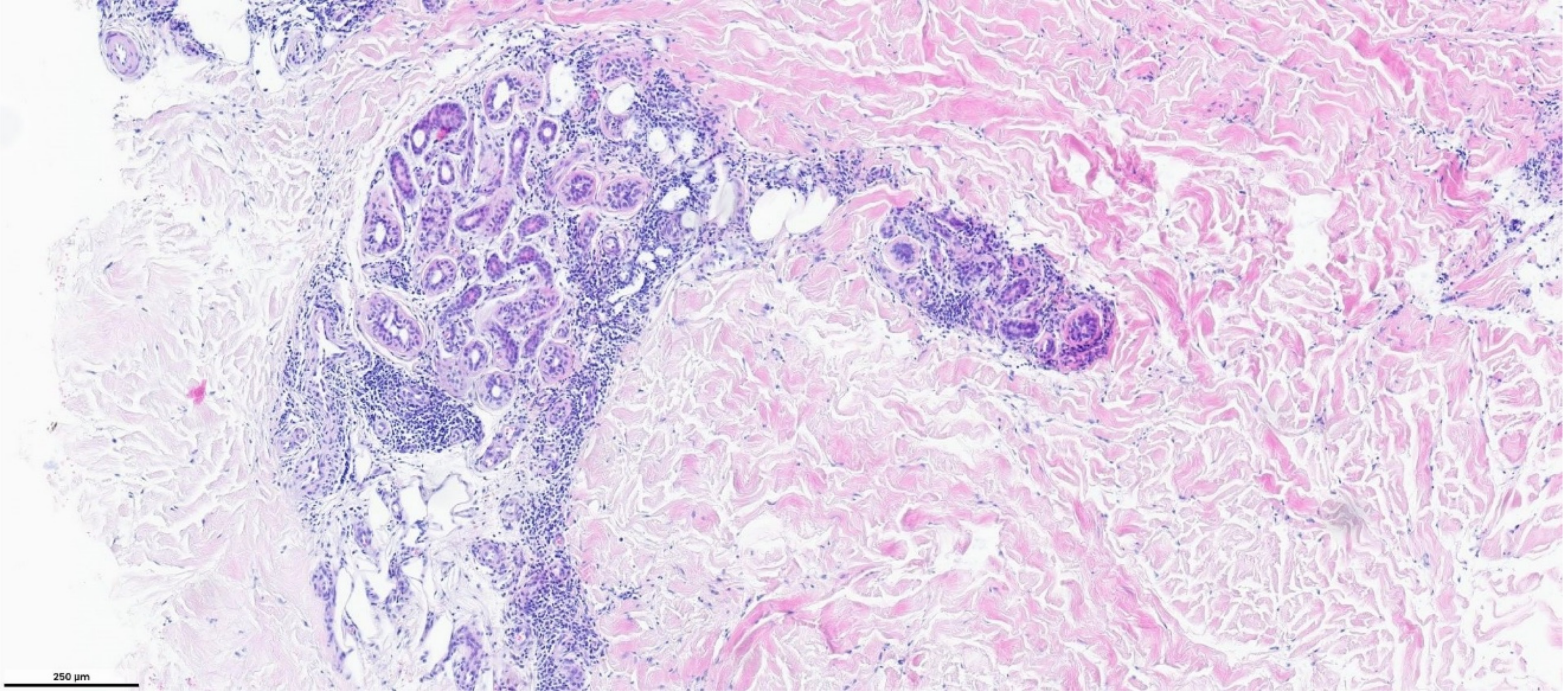
Histopathological section demonstrating perivascular and periadnexal lymphocytic infiltration extending into the deep dermis.

Drug‐induced PLEVA is rare but documented with several medications, including antibiotics (e.g., penicillins and macrolides), vaccines, and immunomodulatory therapies such as tumor necrosis factor inhibitors, though causality often remains uncertain [3]. GLP‐1 receptor agonists have been increasingly associated with immune‐mediated dermatologic conditions, including bullous pemphigoid, eosinophilic panniculitis, morbilliform eruptions, and lichen planus pigmentosus [4, 5]. The pathogenesis of PLEVA involves aberrant T‐cell–mediated immune responses to infectious or pharmacologic triggers [6]. Emerging evidence suggests that tirzepatide exerts immunomodulatory effects through downregulation of NF‐κB, JNK, and NLRP3‐mediated signaling pathways [7]. Although generally beneficial, such immune modulation may paradoxically precipitate inflammatory cutaneous reactions in susceptible individuals.

The close temporal relationship between tirzepatide initiation and lesion onset, along with the absence of alternative precipitating factors, supports a possible drug‐related etiology. Limitations include the absence of a formal causality assessment using the Naranjo Adverse Drug Reaction Probability Scale; the patient chose to discontinue tirzepatide, and rechallenge was therefore not attempted, data that would have strengthened the case for a drug‐induced etiology. Long‐term follow‐up was also unavailable. Nonetheless, this case expands the spectrum of potential cutaneous adverse events associated with tirzepatide. As the use of dual GLP‐1/GIP receptor agonists continues to expand, clinicians should remain vigilant for new‐onset inflammatory skin eruptions following initiation of these agents, as early recognition may guide appropriate management and contribute to post‐marketing safety surveillance.

## Funding

The authors have nothing to report.

## Conflicts of Interest

The authors declare no conflicts of interest.

## Data Availability

The data that support the findings of this study are available from the corresponding author upon reasonable request.
